# Impact of the China-Pakistan Economic Corridor on the China-Europe and China-Middle East trading route selection

**DOI:** 10.1371/journal.pone.0288328

**Published:** 2023-07-13

**Authors:** Khalid Mehmood Alam, Li Xuemei, Saranjam Baig, Faqeer Muhammad, Jingxiao Sun, Muhammad Tariq

**Affiliations:** 1 China Study Centre, Karakoram International University, Gilgit, 15100, Pakistan; 2 School of Economics and Management, Beijing Jiaotong University, Beijing, 100044, China; 3 College of Economics and Political Science, Sultan Qaboos University, Muscat, Oman; 4 School of Economics and Management, Southeast University, Jiangning District, Nanjing, 211189, China; Hosei University: Hosei Daigaku, JAPAN

## Abstract

This research examines the potential impact of the China-Pakistan Economic Corridor (CPEC) on the selection of trading routes between China, the Middle East, and Europe, with a specific focus on the transportation of a 40-foot standard container carrying general commodities. The study compares traditional routes with the new CPEC routes in terms of time, distance, and cost. The findings indicate that the new CPEC routes offer reduced travel time and distance when compared to the traditional routes across all provinces involved. The research reveals that the cost of road transportation along the new CPEC route is lower for Xinjiang province, but higher for the other provinces. By utilizing the new CPEC routes, the time required for goods to travel from China to the Middle East and Europe will be reduced by 10 to 20 days. Furthermore, the distance covered in this trade route will be shortened by 3,000 to 10,000 kilometres. Specifically, the province of Xinjiang in western China stands to benefit significantly from the new CPEC routes, saving approximately $2,000 on trade with the Middle East and Europe. These findings highlight the potential advantages and economic benefits that can be realized by leveraging the CPEC for trade between China, the Middle East, and Europe, particularly in terms of reduced transportation time and distance.

## 1. Introduction

In the wake of the country’s recent reforms, China has surpassed the rest of the world as the world’s primary commerce hub and manufacturing hub [1]. When it comes to trading partners, Europe and the Middle East play crucial roles in China’s export economy. China’s top two export destinations are Europe (18.5%) and the Middle East (15%) [2]. However, China heavily depends on sea transport for foreign trade due to under-developed road and rail connectivity with neighbouring countries [3]. Presently, the sea trade between China, Middle East and Europe are performed from seaports located in South or East China. More than eight thousand nautical miles of water separate the Strait of Malacca from the South China Sea, the Red Sea from the Indian Ocean, the Mediterranean from the Middle East, and the Mediterranean from Europe. As a result, the Strait of Malacca—famous for being a traffic bottleneck due to its unpredictable political climate and massive traffic movement—plays a crucial role in the present marine commercial route from China to the Middle East and Europe [4]. Because of potential roadblocks and regional conflicts, China has serious concerns about the reliability of this sea route, giving rise to the so-called Malacca Dilemma [5]. Another criticism levelled about maritime transport is that it has a poor reputation for mode consistency, is slow, and has unfavourable delivery times.

The presence of these traffic bottlenecks has provided an opportunity for China to review the travel time, cost, and distance of both the traditional trading routes and the new CPEC route to Europe and the Middle East. One of the suggested viable transport routes is the China Pakistan Economic Corridor (CPEC). This route connects the western part of China to Gwadar port in the south of Pakistan. In light of this, the existing research evaluates the efficiency and effectiveness of CPEC as an alternative to the existing trading routes in various provinces of China, connecting them to Europe and the Middle East.

The China-Pakistan Economic Corridor (CPEC) was launched in 2013 by the Chinese government with the intention of increasing the efficiency of international trade and decreasing the impact of existing transport blockades [6]. The goal of the CPEC is to improve the interconnectivity of China’s international logistics network by building and upgrading a variety of road and rail infrastructure. To lessen reliance on the Strait of Malacca, the CPEC will create a potential link between China, the Middle East, and Europe.

Chinese and Pakistani economies will profit greatly from CPEC’s creation. Economically, the growth of the trading market between China, the Middle East, and Europe stands to benefit from the potential for reduced travel distances, reduced delivery times, and reduced costs for Chinese export industries. Despite the obvious progress, the CPEC continues to encounter obstacles. There are many unknowns regarding the safety of the transportation networks that make up the China–Pakistan economic corridor. Collaboration contracts on transportation infrastructure development, upgrading, or pass-through permission, for example, may be put on hold or even terminated if there is a change in government or government policies.

The existing literature rarely focus on cost and benefit analysis of the traditional and new CPEC route and how the CPEC route can function as a substitute for the traditional route between China, Middle East, and Europe. Likewise, only a few studies have, to our knowledge, statistically examined the varying implications of the CPEC on the trading route decisions of the industries based in different provinces/municipalities of China. To assess the benefits of traditional and new CPEC route, in this research, we have statistically compared three variables, namely time, distance and cost, which present important policy implications for both policy makers and those associated with industries.

Instead of focusing on evaluating national infrastructure choices, we will be focusing on the benefits of CPEC as an alternate route to the traditional route for international trade between China, the Middle East, and Europe.

### 1.1 Importance of the trading route selection issue

For the trade market between China, the Middle East, and Europe, CPEC presents both huge prospects and major problems. And because CPEC has so many unique features, it will have varying effects in various regions of China. To stay profitable in the rapidly expanding, fiercely competitive export market in China, businesses must carefully consider the time, distance, and cost of each possible trading route. Although the significance of the trading route selection problem is recognised, there is scant research available to analyse the unique features of CPEC and logically investigate their contradictory consequences on the decision making of Chinese exporting enterprises. As a result, decision-makers are not fully apprised of the CPEC’s merits and pitfalls, leading to insufficient investment and limited success.

The next section presents the literature review. Section 3 presents the methodology to estimate the transport cost, distance, and travel time. Section 4 presents the results and conclusion followed by section five.

## 2. Literature review

The existing literature that is related to the China Pakistan Economic Corridor generally investigates the economic and political implications as well as the motivations behind it. Few studies, however, have investigated the specifics of the economic corridors proposed by the BRI and their viability as alternate routes for trade between China and the Middle East and Europe.

For research methodology and statistical analysis, this research paper relies on [7], who examine China Pakistan Economic Corridor’s impact on transport cost and travel time and descriptive statistical approaches were used. Transport cost and travel time are computed and compared for the traditional and new CPEC routes. The results suggest that the transport cost for 40-foot container from Kashgar to Middle east is reduced by $1450 and Kashgar to Europe is reduced by $1350. The travel time from Kashgar to Middle east and Europe is decreased by 21 to 24 days.

Unlike [7], which considers only Kashgar city in Xinjiang province for comparison of transport cost, travel time and distance, the current research considers all the provinces and municipalities in China for comparison of transport cost, travel time and distance between traditional route and new CPEC route.

Some existing literature (for instance, [8–10]) provide a thorough classification and description of intermodal freight transport, which has gained a lot of attention from researchers over the past two decades. [11] classified land bridges as either "land bridges," "mini bridges," or "microbridges" based on their endpoints and starting points. Some research [12–16] also investigated the potential and value of land bridges for multimodal transport. The CPEC road and rail projects were jointly begun by China and Pakistan to build an intermodal transport system, and China uses Pakistan as a land bridge to ship its goods to the Middle East and Europe. Land connections were emphasised by [17–19] as a means of diverting freight away from main thoroughfares. The importance of intermodal transport service in reducing freight transportation costs, reducing transit times, and shortening routes was also discussed in this research. When speed is of the essence and must take precedence over other considerations, various intermodal freight transit options are kept in mind [20].

The U.S. serves as a land bridge to allow ships to avoid the Panama Canal [21]. This research indicates that bigger ships would take the Panama Canal instead of the land bridge to save time. A combination of smaller ships, vehicles, and trains will complete the transcontinental journey of the containers when they are unloaded at west coast ports. A 590-mile double-track railroad from Jeddah to Dammam is now under construction in Saudi Arabia, connecting the country’s Red Sea and Gulf coasts. Currently, transportation between these ports takes roughly three days. The time it takes to go from port to port will be cut by roughly 10 hours thanks to the new train connection [22,23]. The "Taiwan land bridge project," which involves a world-class port and a dry port connected by railway and motorways, is being developed by the Thai government. The concept is modelled after the Eastern Seaboard project. The Thailand land bridge will shorten the time it takes to move cargo and will allow it to avoid the Strait of Malacca [24]. The Great Equatorial Land Bridge will connect Douala, Cameroon, to Lamu, Kenya, by way of the Central African Republic and South Sudan. This proposed rail land bridge between the South Atlantic and Indian oceans would be around 2625 miles in length. The average speed of the cargo trains will be 75 miles per hour [25], and they will be able to transport 20 million TUEs each year [26].

The time and money needed to transport commodities from China to Europe across the Eurasian land bridge were compared by [27]. The research found that the time it takes to transport goods through land bridge is ten days less than when using the sea [28]. The effects on freight transportation when railways are used instead of the Panama Canal to cross the United States were studied by [29]. Under typical conditions, a ship can only make 20–25 knots, which translates to less than 20 miles per hour. The time needed to travel a distance is cut by five or six days because to the superior speed of trains compared to ships. [30] identified the obstacles that impede effective use of the Eurasian land bridge, such as the imperfect operation of trains and ports on the land bridge.

A study by [31] evaluated the transportation costs of one TEU for four distinct intermodal routes. The results showed that high logistic expenses and poor physical infrastructure have not been adapted to modern intermodal business practises. To increase confidence in projects for better resource allocation, [32] created a multiperiod dynamic model. This methodology will aid managers in determining how much resources should be allocated to bolster process reliability. To reduce the firm’s failure cost and preventative cost, the best allocation of investments will be made. The research indicates that investment will be done on a regular basis if output growth is expected to be substantial, for a shorter time frame if growth is expected to be intermediate, and all at once if growth is expected to be little.

Export performance was studied by [33,34], who investigated the role that infrastructure quality played. The findings show that reducing the exporter’s transport costs by investing in better infrastructure has a beneficial effect on exports. Furthermore, the findings imply that the minimal quality of infrastructure between two trading countries is the most important factor in transportation costs and commerce.

According to the research that was conducted by [35] in their bilateral trade model that included transport expenses, it is believed that the costs of transportation are inversely proportional to the amount of infrastructure. [36,37] conducted research to investigate the effect that time gaps have on international business transactions. According to the findings, the amount of international trade is reduced by at least one percent for each day that a product is delayed prior to shipment [38]. This means that each day is equivalent to a nation moving away from its trade partners by an average of seventy kilometres [39–41] conducted research to investigate how a nation’s trade performance is affected by the quality of its infrastructure.

## 3. Methodology

The purpose of this section is to demonstrate the effect of the China-Pakistan Economic Corridor by evaluating the impacts of the China-Pakistan Economic Corridor on the trading route selection between China and the Middle East and Europe. The evaluation is based on the delivery of a standard container measuring 40 feet in length containing a general good.

In this study, the transport costs, travel time, and distance are estimated and compared for the new CPEC route as well as the traditional route. These two itineraries are based on both land and seaways. A detailed description of travel time, cost and distance variables have been provided in the following sections. Given the nature and availability of data, using an advance econometric estimation technique was not possible. The data collected was scattered and discrete. Considering these limitations this research followed a simple statistical analysis using MS excel. The secondary data on travel time, cost and distance was collected from various online sources including shipping corporations, google maps and transportation companies. The data gathered was then cleaned and arranged systematically to be analysed in MS excel.

### 3.1 Product origin

China, which is the third largest country in the world and has an economy that is rapidly increasing. Instead of focusing on the evaluation of various national infrastructures, the primary objective of this study is to investigate the potential benefits of the China-Pakistan Economic Corridor (CPEC) over the more conventional route in international trade between China, the Middle East, and Europe.

Table 1 contains information regarding China’s most important ports located in each of the country’s provinces/municipalities. The major ports are presented in the third column, while the different provinces and municipalities are listed in the second column. This study considers Shanghai port, Tianjin port, Qingdao port, Guangzhou port, Ningbo port, and Dalian port as China’s six largest ports [2]. Zhejiang province is near the provinces of Fujian and Jiangxi. As a result, the three provinces in question conduct their commercial activities in the port of Ningbo, which is located on the coast of the East China Sea. In a similar manner, considering the locations of the country’s other main seaports, the provinces of Guangdong, Guangxi, and Hainan should do business via the Guangzhou Port, whilst the provinces of Shandong and Henan should conduct business via the Qingdao Port.

**Table 1:** List of selected provinces and municipalities with major seaports.

S. No	Province/Municipality	Major Sea Port
1	Xinjiang	Shanghai Port
2	Tibet	Shanghai Port
3	Inner Mongolia	Shanghai Port
4	Guangdong	Guangzhou Port
5	Guangxi	Guangzhou Port
6	Hainan	Guangzhou Port
7	Shanghai	Shanghai Port
8	Jiangsu	Shanghai Port
9	Anhui	Shanghai Port
10	Zhejiang	Ningbo Port
11	Fujian	Ningbo Port
12	Jiangxi	Ningbo Port
13	Shandong	Qingdao Port
14	Henan	Qingdao Port
15	Tianjin	Tianjin Port
16	Beijing	Tianjin Port
17	Hebei	Tianjin Port
18	Liaoning	Dalian Port
19	Jilin	Dalian Port
20	Heilongjiang	Dalian Port
21	Qinghai	Shanghai Port
22	Gansu	Shanghai Port
23	Ningxia	Shanghai Port
24	Shanxi	Shanghai Port
25	Shaanxi	Shanghai Port
26	Sichuan	Shanghai Port
27	Chongqing	Shanghai Port
28	Yunnan	Shanghai Port
29	Guizhou	Shanghai Port
30	Hunan	Shanghai Port
31	Hubei	Shanghai Port

**Fig 1:**
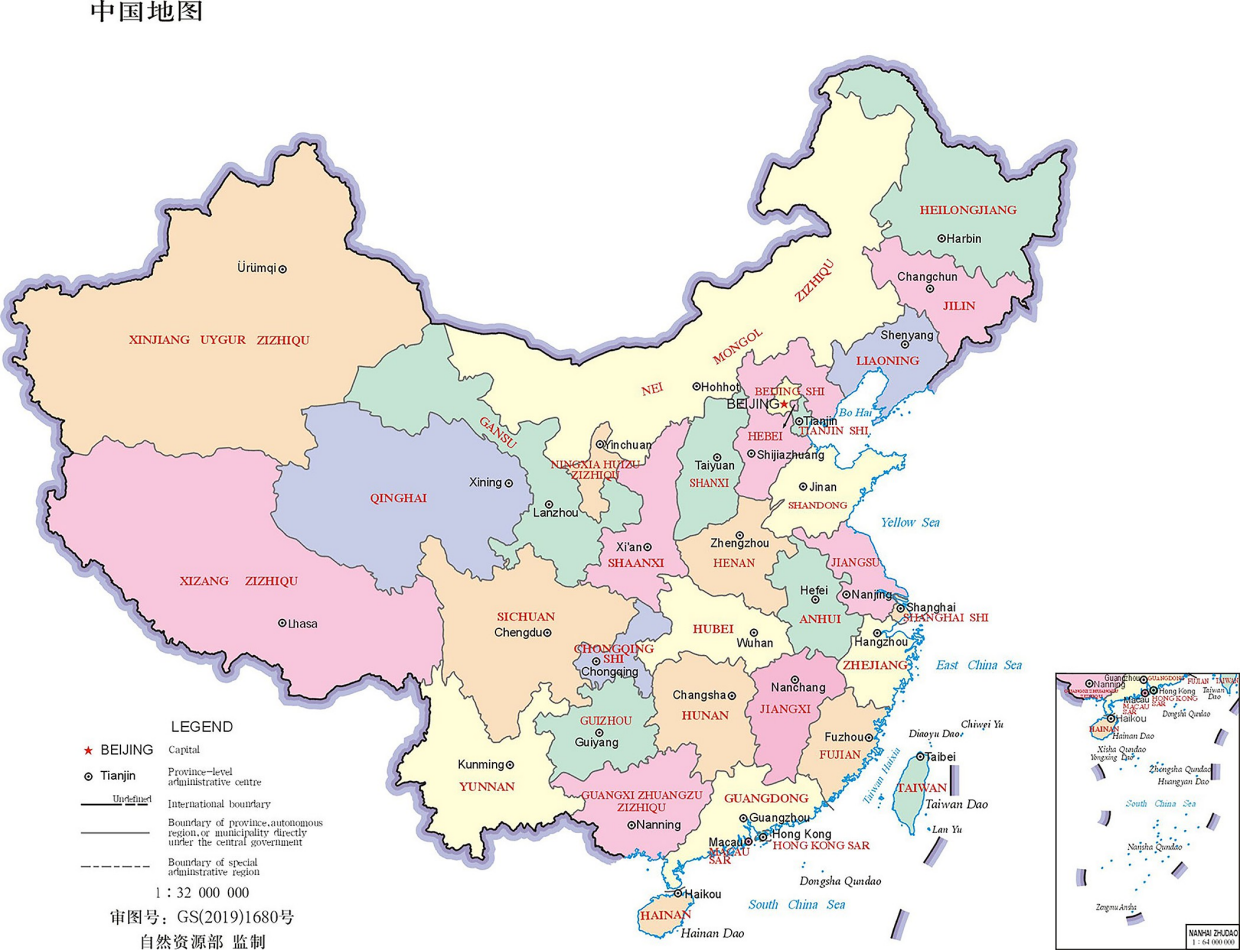
Provinces and municipalities of China.

**Fig 2:**
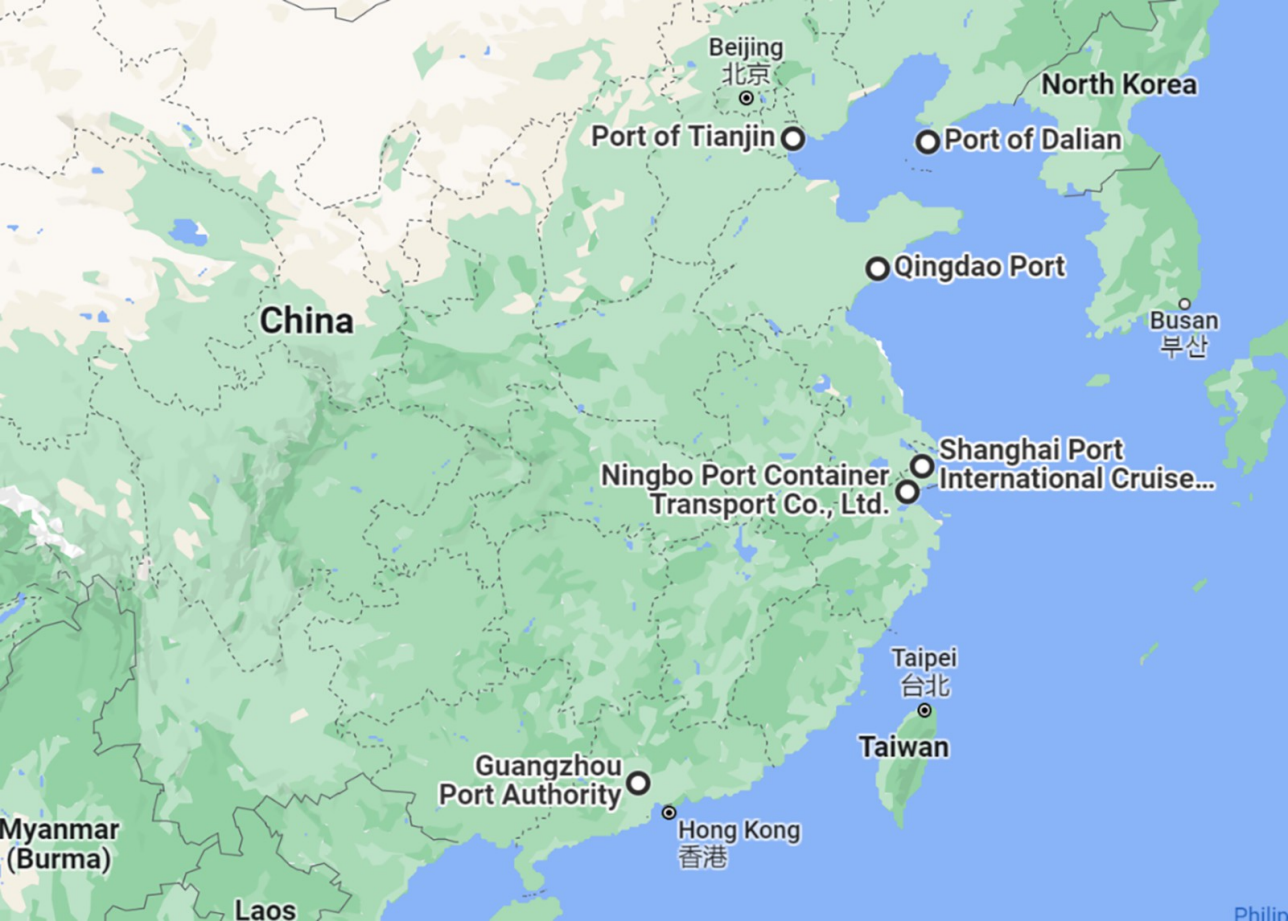
Major Sea Ports in China.

Likewise, Tianjin, Beijing and Hebei municipalities will use Tianjin Port, and Liaoning, Jilin and Heilongjiang provinces will trade via Dalian Port. Lastly, Shanghai, Jiangsu, Anhui and the inland non-coastal provinces and municipalities like Xinjiang, Tibet, Inner Magnolia, Qinghai, Gansu, Ningxia, Shanxi, Shaanxi, Sichuan, Chongqing, Yunnan, Guizhou, Hunan, and Hubei will select the Port of Shanghai which is the largest seaport in the China for trade. Fig 1 presents the provinces and municipalities while Fig 2 depicts the major seaports of China.

### 3.2 Destination

In this research, United Arab Emirates (UAE) in Middle East and Germany in Europe are selected as export destination countries.

When looking at European countries, Germany is by far the most important market for Chinese exports. It has been revealed that China has shipped items to Germany worth 115.18 billion USD in the year 2021. In addition, the Port of Hamburg is a universal port, which means it can handle all different sorts of cargo. It provides a variety of services, including those for the handling of goods, the passage of customs, quality control, storage and packing, and distribution. It is the "Gateway to the World" for Germany because it is the largest port in terms of volume, and it is in Germany. As a direct consequence of this finding, the Port of Hamburg has been decided upon as the location of the product’s destination.

**Fig 3:**
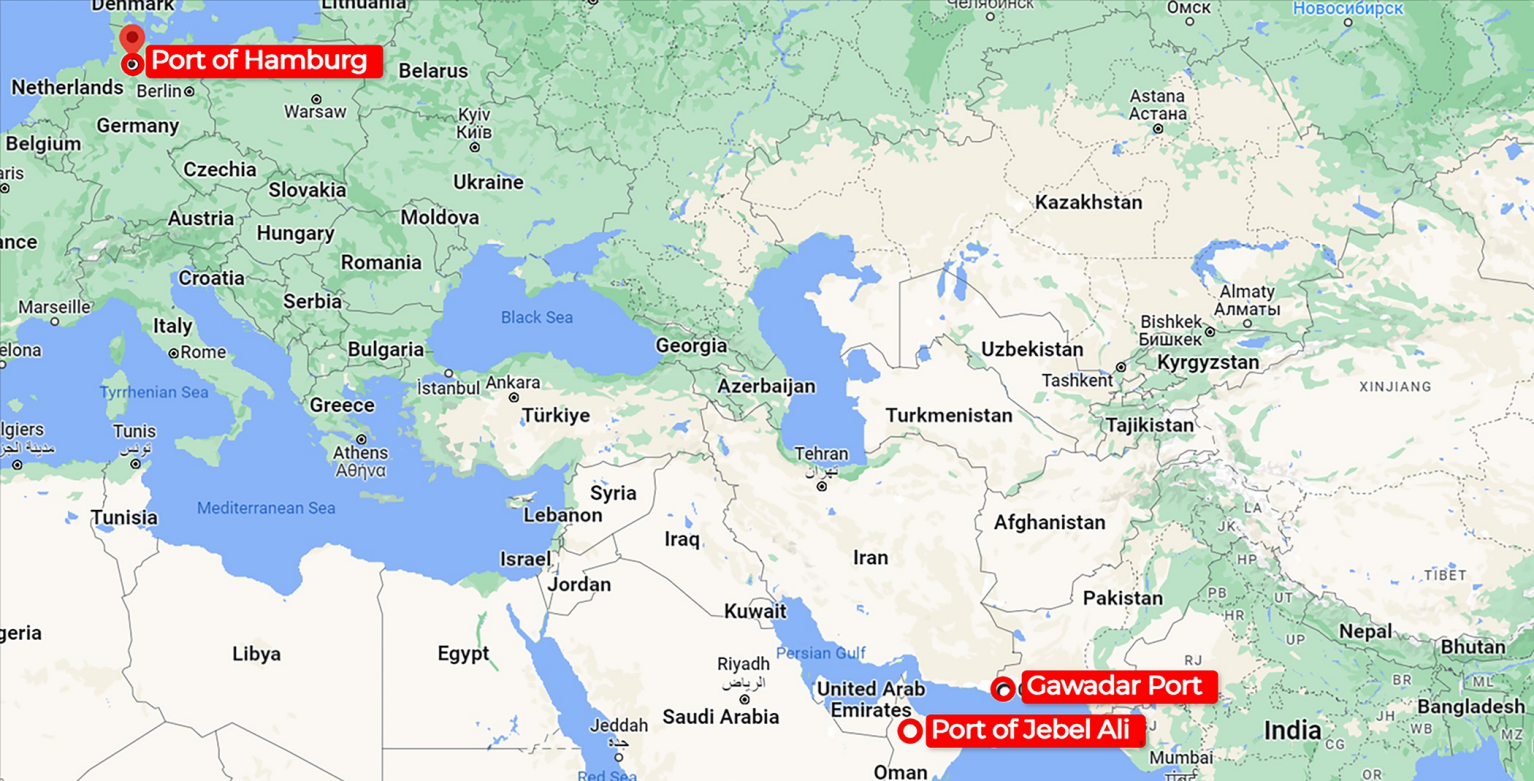
Destination Ports.

United Arab Emirates is the largest trade market among Middle East countries. It is reported that China has exported 5.26 billion USD value of goods to UAE and imported $3.43B value of goods from United Arab Emirates. UAE trades mostly via Port of Jebel Ali. It is the world’s nineth busiest port, the largest man-made harbour, and the biggest and by far the busiest port in the Middle East. Jebel Ali Port has been voted “Best Seaport–Middle East” for 24 consecutive years. As an integrated multi-modal hub offering sea, air, and land connectivity, complemented by extensive logistics facilities, the Port plays a vital role in the UAE economy. As a result, Port of Jebel Ali is considered as the product destination in our analysis. Fig 3 presents the destination ports.

### 3.3 Traditional route

**Fig 4:**
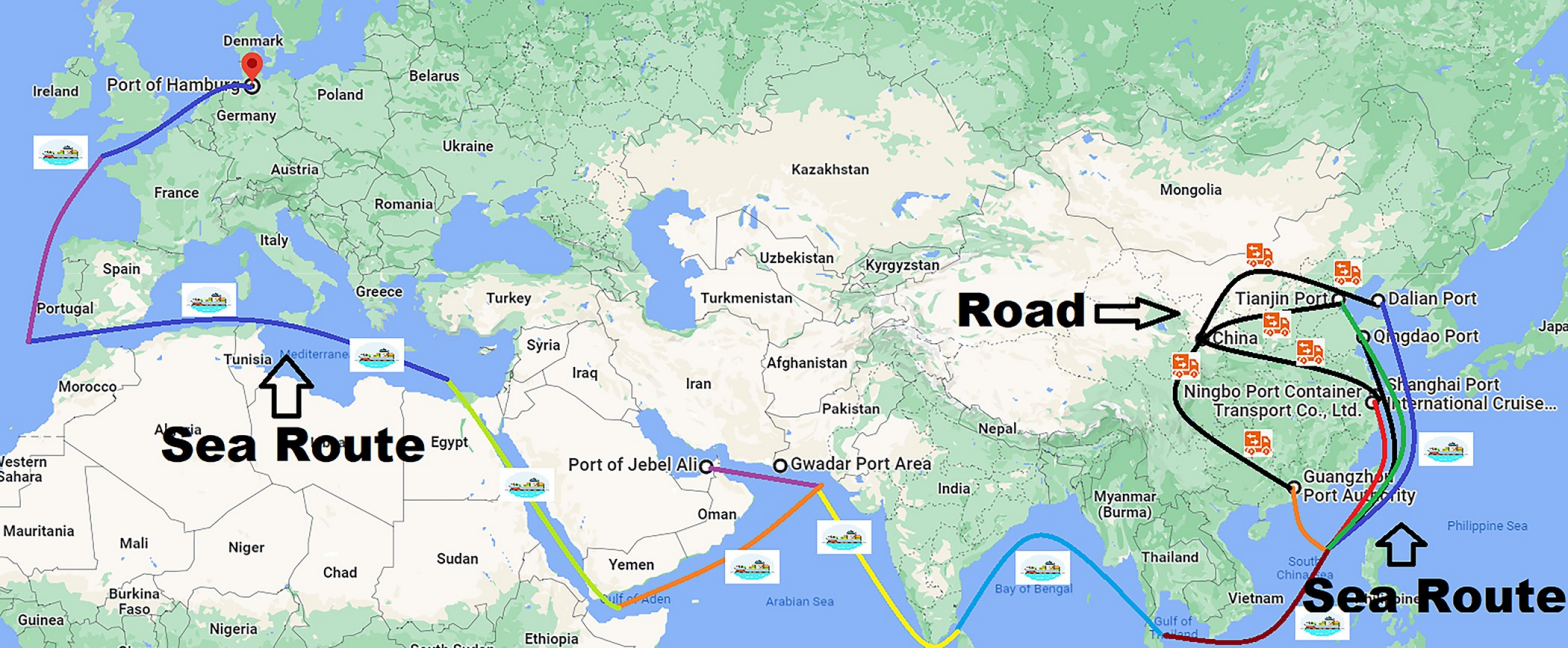
Traditional Route.

The traditional route from different provinces/municipalities in China to selected destination port in Europe and the Middle East comprises of a seaway and a roadway. The traditional route is divided into two parts, the distance from destination province/municipality to selected seaport in China is called a roadway, and distance from selected seaport to Port of Hamburg in Germany and Port of Jebel Ali in Middle East is called a seaway. In this section, the research paper estimates the transport cost, distance, and travel time for a 40-foot container that is transported from different provinces/municipalities of China to Port of Hamburg in Germany and Port of Jebel Ali in United Arab Emirates. The 40 feet container for general products comes from different provinces/municipalities to designated ports in China by road, which are transported to the destination seaports in Germany and UAE by sea. Fig 4 shows the traditional, which China to trade from China to Middle East and Europe.

#### 3.3.1 Travel time

Travel time for road transportation is attained by dividing total distance by average speed of a truck. For the purposes of this study, we will assume that the truck travels at a constant speed of 40 kilometres per hour (kph), even if its maximum speed may reach 80 kilometres per hour (km/h) on a flat road and its minimum speed may fall to 30 km/h (km/h) or below in mountainous regions. Total distance is retrieved from Google Maps. Travel time for sea is obtained by using an online software ports.com [42]. The average speed of 12 knots is used to determine the travel time for cargo ship. Taking 12 knots as the standard speed of ships has huge benefits for humans, nature, and the climate. As ships travel more slowly, they burn less fuel, which means there are also savings in black carbon, sulphur, and nitrogen oxides. The existing literature found that reducing the speed of ship by 20% would reduce sulphur and nitrogen oxides by around 24%. and cut underwater noise by 66% and reduce the chances of whale collisions by 78% [43].

Travel time estimations for traditional trading route are presented in Table 2. Column 4 of Table 2 presents the travel time for road from different provinces/municipalities to designated ports in China. For example, a 40 feet container from Kashgar will take four days by road to reach to shanghai port while a container from Zhejiang province will take just 0.1 days to reach to Ningbo port. Column 5 shows the travel time for sea from different ports of China to Port of Jebel Ali in UAE. A cargo ship will take 25.5 days from shanghai port to Port of Jebel Ali while a cargo ship will reach in 22.6 days from Guangzhou port. Column 6 shows the total travel time including road and sea to Port of Jebel Ali in UAE from designated ports in China. A container from Xinjiang will reach in 29.6 days to Port of Jebel Ali in UAE while a 40 feet container will reach to UAE in 22.6 days from Guangdong province. Likewise, a 40 feet container from Xinjiang will reach in 46.7 days to Germany. Details for all remaining provinces have been provided in the Table 2.

**Table 2:** Traditional route travel time.

(1) S. No	(2) Province/ Municipality	(3) Major Sea Port	(4) Road travel time (From Province/ Municipality to destination Port in China)	(5) Sea travel time (Destination Port in China to UAE)	(4 + 5) = (6) Total travel time (UAE)	(7) Sea travel time (Germany)	(4 + 7) = (8) Total travel time (Germany)
1	Xinjiang	Shanghai Port	4.1 days	25.5 days	29.6 days	42.6 days	46.7 days
2	Tibet	Shanghai Port	4.1 days	25.5 days	29.6 days	42.6 days	46.7 days
3	Inner Mongolia	Shanghai Port	1.8 days	25.5 days	27.3 days	42.6 days	44.4 days
4	Guangdong	Guangzhou Port	0.1 days	22.6 days	22.7 days	39.7 days	39.8 days
5	Guangxi	Guangzhou Port	0.7 days	22.6 days	23.3 days	39.7 days	40.4 days
6	Hainan	Guangzhou Port	0.6 days	22.6 days	23.2 days	39.7 days	40.3 days
7	Shanghai	Shanghai Port	0.0 days	25.5 days	25.5 days	42.6 days	42.6 days
8	Jiangsu	Shanghai Port	0.3 days	25.5 days	25.8 days	42.6 days	42.9 days
9	Anhui	Shanghai Port	0.5 days	25.5 days	26.0 days	42.6 days	43.1 days
10	Zhejiang	Ningbo Port	0.1 days	25.3 days	25.4 days	42.4 days	42.5 days
11	Fujian	Ningbo Port	0.7 days	25.3 days	26.0 days	42.4 days	43.1 days
12	Jiangxi	Ningbo Port	0.7 days	25.3 days	26.0 days	42.4 days	43.1 days
13	Shandong	Qingdao Port	0.4 days	26.7 days	27.1 days	43.9 days	44.3 days
14	Henan	Qingdao Port	0.7 days	26.7 days	27.4 days	43.9 days	44.6 days
15	Tianjin	Tianjin Port	0.1 days	28.1 days	28.2 days	45.2 days	45.3 days
16	Beijing	Tianjin Port	0.2 days	28.1 days	28.3 days	45.2 days	45.4 days
17	Hebei	Tianjin Port	0.4 days	28.1 days	28.5 days	45.2 days	45.6 days
18	Liaoning	Dalian Port	0.4 days	27.5 days	27.9 days	44.6 days	45.0 days
19	Jilin	Dalian Port	0.8 days	27.5 days	28.3 days	44.6 days	45.4 days
20	Heilongjiang	Dalian Port	1.0 days	27.5 days	28.5 days	44.6 days	45.6 days
21	Qinghai	Shanghai Port	2.4 days	25.5 days	27.9 days	42.6 days	45.0 days
22	Gansu	Shanghai Port	2.1 days	25.5 days	27.6 days	42.6 days	44.7 days
23	Ningxia	Shanghai Port	2.1 days	25.5 days	27.6 days	42.6 days	44.7 days
24	Shanxi	Shanghai Port	1.4 days	25.5 days	26.9 days	42.6 days	44.0 days
25	Shaanxi	Shanghai Port	1.5 days	25.5 days	27.0 days	42.6 days	44.1 days
26	Sichuan	Shanghai Port	2.1 days	25.5 days	27.6 days	42.6 days	44.7 days
27	Chongqing	Shanghai Port	1.8 days	25.5 days	27.3 days	42.6 days	44.4 days
28	Yunnan	Shanghai Port	2.5 days	25.5 days	28.0 days	42.6 days	45.1 days
29	Guizhou	Shanghai Port	1.9 days	25.5 days	27.4 days	42.6 days	44.5 days
30	Hunan	Shanghai Port	1.2 days	25.5 days	26.7 days	42.6 days	43.8 days
31	Hubei	Shanghai Port	0.9 days	25.5 days	26.4 days	42.6 days	43.5 days

#### 3.3.2 Distance

Google Maps, an online mapping tool, was used to calculate the distance travelled by land, while ports.com was used to calculate the sea distance. To calculate the total distance of the traditional route, sea distance and land distance are added together. The length of the sea journey is expressed in nautical miles, which may be translated to kilometres by multiplying the value by 1.852.

Table 3 presents the total distance for traditional route from different provinces/municipalities of China to destination ports in UAE and Germany. Column 4 shows the road distance in kilometres from different provinces/municipalities to designated ports in China. For example, a road distance from Kashgar to shanghai port is 3950 kms while a distance from Zhejiang province to Ningbo port is 110 kms. Column 5 shows the sea distance from different ports of China to Port of Jebel Ali in UAE. A sea distance from shanghai port to Port of Jebel Ali in UAE is 13596 kms while sea distance from Guangzhou port is 12053 kms. Column 6 shows the total distance including road and sea to Port of Jebel Ali in UAE from different provinces/municipalities in China. A total distance from Xinjiang to Port of Jebel Ali in UAE is 17546 kms while a total distance from Guangdong Province is 12139 kms. Column 8 shows the total distance including road and sea to Hamburg port in Germany from different provinces/municipalities of China. For example, a total distance from Xinjiang to Germany is 26687 kms while a total distance from Guangdong Province is 21280 kms. Details for all remaining provinces have been provided in the Table 3.

**Table 3:** Traditional route distance.

(1) S. No	(2) Province/ Municipality	(3) Major Sea Port	(4) Road distance in Kms (From Province/ Municipality to destination Port in China)	(5) Sea distance kms (Destination Port of China to UAE)	(4+5) = (6) Total Distance (China to UAE)	(7) Sea distance kms (Destination Port of China to Germany)	(4+7) = (8) Total Distance kms (China to Germany)
1	Xinjiang	Shanghai Port	3950	13596	17546	22737	26687
2	Tibet	Shanghai Port	3954	13596	17550	22737	26691
3	Inner Mongolia	Shanghai Port	1748	13596	15344	22737	24485
4	Guangdong	Guangzhou Port	86	12053	12139	21194	21280
5	Guangxi	Guangzhou Port	674	12053	12727	21194	21868
6	Hainan	Guangzhou Port	618	12053	12671	21194	21812
7	Shanghai	Shanghai Port	5	13596	13601	22737	22742
8	Jiangsu	Shanghai Port	312	13596	13908	22737	23049
9	Anhui	Shanghai Port	467	13596	14063	22737	23204
10	Zhejiang	Ningbo Port	110	13499	13609	22641	22751
11	Fujian	Ningbo Port	702	13499	14201	22641	23343
12	Jiangxi	Ningbo Port	672	13499	14171	22641	23313
13	Shandong	Qingdao Port	341	14247	14588	23389	23730
14	Henan	Qingdao Port	677	14247	14924	23389	24066
15	Tianjin	Tianjin Port	57	14977	15034	24119	24176
16	Beijing	Tianjin Port	172	14977	15149	24119	24291
17	Hebei	Tianjin Port	395	14977	15372	24119	24514
18	Liaoning	Dalian Port	402	14651	15053	23791	24193
19	Jilin	Dalian Port	787	14651	15438	23791	24578
20	Heilongjiang	Dalian Port	931	14651	15582	23791	24722
21	Qinghai	Shanghai Port	2259	13596	15855	22737	24996
22	Gansu	Shanghai Port	2015	13596	15611	22737	24752
23	Ningxia	Shanghai Port	1989	13596	15585	22737	24726
24	Shanxi	Shanghai Port	1377	13596	14973	22737	24114
25	Shaanxi	Shanghai Port	1426	13596	15022	22737	24163
26	Sichuan	Shanghai Port	1969	13596	15565	22737	24706
27	Chongqing	Shanghai Port	1701	13596	15297	22737	24438
28	Yunnan	Shanghai Port	2389	13596	15985	22737	25126
29	Guizhou	Shanghai Port	1840	13596	15436	22737	24577
30	Hunan	Shanghai Port	1119	13596	14715	22737	23856
31	Hubei	Shanghai Port	832	13596	14428	22737	23569

#### 3.3.3 Cost

The traditional route cost is estimated by adding both sea and road cost. Total cost of road transportation is estimated by multiplying the per kilometre cost with total kilometres. Different transporters charge different per kilometre cost, so average per kilometre cost is used to determine the road cost. Following the analysis of [7], the average per kilometre cost of 0.5$ is taken in our analysis to determine the road transport cost. Cost of sea is taken from website of Hapag-Lloyd [44]. Hapag-Lloyd AG is a German international shipping and container transportation company which was formed in 1970. It’s important to note that these costs are valid only for the month of June 2022.

Table 4 presents the total cost for traditional route from different provinces/municipalities of China to destination ports in UAE and Germany. Column 4 shows the road cost in US dollars from different provinces/municipalities to designated ports in China. For example, a road cost from Kashgar to shanghai port is 1975 dollars while a road cost from Zhejiang province to Ningbo port is 55 dollars. Column 5 shows the sea cost from different ports of China to Port of Jebel Ali in UAE. A sea cost from shanghai port to Port of Jebel Ali in UAE is 3368 US dollars while sea cost from Guangzhou port is 4768 US dollars. Column 6 shows the total cost including road and sea to Port of Jebel Ali in UAE from different provinces/municipalities in China. A total cost of 40 feet container from Xinjiang to Port of Jebel Ali in UAE is 5343 US dollars while a total cost from Guangdong Province is 4811 US dollars. Column 8 shows the total cost including road and sea to Hamburg port in Germany from different provinces/municipalities of China. A total cost of 40 feet container from Xinjiang to Germany is 9881 US dollars while a total cost from Guangdong Province is 7949 US dollars. Details for all remaining provinces have been provided in the Table 4.

**Table 4:** Traditional route cost.

(1) S. No	(2) Province/ Municipality	(3) Major Sea Port	(4) Road Cost in $ (From Province/ Municipality to destination Port in China)	(5) Sea Cost in $ (Destination Port in China to UAE)	(4+5) = (6) Total cost in $ (China to UAE)	(7) Sea cost in $ (Destination Port to Germany)	(4+7) = (8) Total cost (China to Germany)
1	Xinjiang	Shanghai Port	1975	3368	5343	7906	9881
2	Tibet	Shanghai Port	1977	3368	5345	7906	9883
3	Inner Mongolia	Shanghai Port	874	3368	4242	7906	8780
4	Guangdong	Guangzhou Port	43	4768	4811	7906	7949
5	Guangxi	Guangzhou Port	337	4768	5105	7906	8243
6	Hainan	Guangzhou Port	309	4768	5077	7906	8215
7	Shanghai	Shanghai Port	3	3368	3371	10341	10344
8	Jiangsu	Shanghai Port	156	3368	3524	10341	10497
9	Anhui	Shanghai Port	234	3368	3602	10341	10575
10	Zhejiang	Ningbo Port	55	3868	3923	7906	7961
11	Fujian	Ningbo Port	351	3868	4219	7906	8257
12	Jiangxi	Ningbo Port	336	3868	4204	7906	8242
13	Shandong	Qingdao Port	171	3484	3655	7906	8077
14	Henan	Qingdao Port	339	3484	3823	7906	8245
15	Tianjin	Tianjin Port	29	3584	3613	7906	7935
16	Beijing	Tianjin Port	86	3584	3670	7906	7992
17	Hebei	Tianjin Port	198	3584	3782	7906	8104
18	Liaoning	Dalian Port	201	3584	3785	7906	8107
19	Jilin	Dalian Port	394	3584	3978	7906	8300
20	Heilongjiang	Dalian Port	466	3584	4050	7906	8372
21	Qinghai	Shanghai Port	1130	3368	4498	7906	9036
22	Gansu	Shanghai Port	1008	3368	4376	7906	8914
23	Ningxia	Shanghai Port	995	3368	4363	7906	8901
24	Shanxi	Shanghai Port	689	3368	4057	7906	8595
25	Shaanxi	Shanghai Port	713	3368	4081	7906	8619
26	Sichuan	Shanghai Port	985	3368	4353	7906	8891
27	Chongqing	Shanghai Port	851	3368	4219	7906	8757
28	Yunnan	Shanghai Port	1195	3368	4563	7906	9101
29	Guizhou	Shanghai Port	920	3368	4288	7906	8826
30	Hunan	Shanghai Port	560	3368	3928	7906	8466
31	Hubei	Shanghai Port	416	3368	3784	7906	8322

### 3.4 New CPEC route

**Fig 5:**
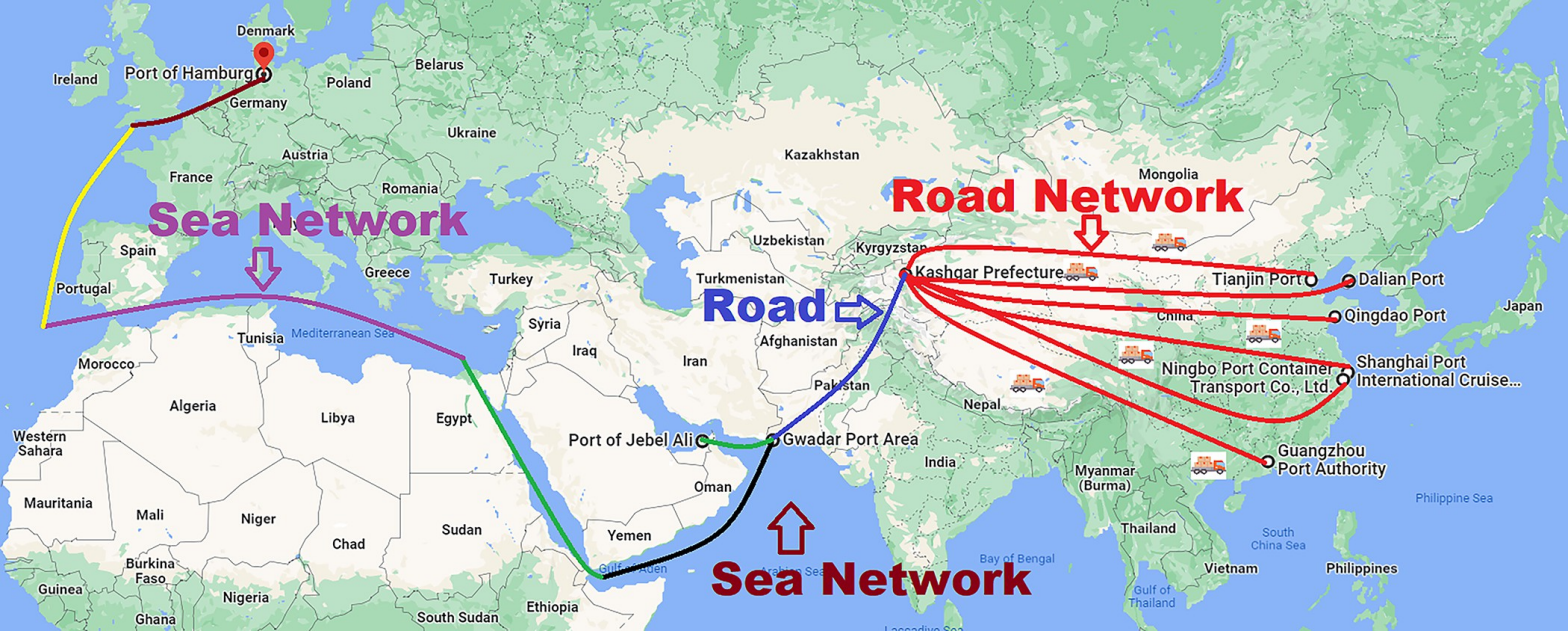
New CPEC route.

The new CPEC route from China to selected destination ports in Europe and the Middle East comprise of a seaway and a roadway. New CPEC route is divided into two sections, the distance from different provinces/municipalities of China to Gwadar port is called a roadway, and distance from Gwadar seaport to port of Jebel Ali in Middle East and Port of Hamburg in Europe is called a seaway. In this section, the research paper calculates the transport cost and travel time for a 40-foot container that is transported by new CPEC route from different provinces/municipalities in China to selected port in Middle East and Europe. The container from different provinces/municipalities in China comes to the Gwadar seaport (Pakistan) by road and goes to destination seaport by sea. Fig 5 shows the new CPEC route, which China will use as an alternative route to trade from China to Middle East and Europe.

#### 3.4.1 Travel time

Travel time estimations for new CPEC route are presented in Table 5. Column 4 presents the travel time for road from different provinces/municipalities to designated ports in China. For example, a 40 feet container from Kashgar will take 2.9 days by road to reach Gwadar port in Pakistan while a container from Zhejiang province will take 8.2 days to reach Gwadar Port in Pakistan. Column 5 shows the travel time for sea from Gwadar port to Port of Jebel Ali in UAE. For instance, a cargo ship will take 7.4 days from Gwadar port to Port of Jebel Ali in UAE. Column 6 shows the total travel time including road and sea to Port of Jebel Ali in UAE from different provinces/municipalities of China. A container from Xinjiang will reach in 10.3 days to Port of Jebel Ali in UAE while a 40 feet container will reach UAE in 15.9 days from Guangdong province. Likewise, a 40 feet container from Xinjiang will reach in 27.4 days to Germany. Details for all remaining provinces have been provided in the Table 5.

**Table 5:** New CPEC route travel time.

(1) S. No	(2) Province/ Municipality	(3) Major Sea Port	(4) Road travel time (Destination Port of China to Gwadar Port)	(5) Sea travel time (Gwadar Port to UAE)	(4 + 5) = (6) Total travel time (UAE)	(7) Sea travel time (Gwadar Port to Germany)	(4 + 7) + (8) Total travel time (Germany)
1	Xinjiang	Shanghai Port	2.9 days	7.4 days	10.3 days	24.5 days	27.4 days
2	Tibet	Shanghai Port	6.4 days	7.4 days	13.8 days	24.5 days	30.9 days
3	Inner Mongolia	Shanghai Port	7.1 days	7.4 days	14.5 days	24.5 days	31.6 days
4	Guangdong	Guangzhou Port	8.5 days	7.4 days	15.9 days	24.5 days	33.0 days
5	Guangxi	Guangzhou Port	8.3 days	7.4 days	15.7 days	24.5 days	32.8 days
6	Hainan	Guangzhou Port	8.9 days	7.4 days	16.3 days	24.5 days	33.4 days
7	Shanghai	Shanghai Port	8.2 days	7.4 days	15.6 days	24.5 days	32.7 days
8	Jiangsu	Shanghai Port	7.9 days	7.4 days	15.3 days	24.5 days	32.4 days
9	Anhui	Shanghai Port	7.8 days	7.4 days	15.2 days	24.5 days	32.3 days
10	Zhejiang	Ningbo Port	8.2 days	7.4 days	15.6 days	24.5 days	32.7 days
11	Fujian	Ningbo Port	8.5 days	7.4 days	15.9 days	24.5 days	33.0 days
12	Jiangxi	Ningbo Port	8.0 days	7.4 days	15.4 days	24.5 days	32.5 days
13	Shandong	Qingdao Port	7.4 days	7.4 days	14.8 days	24.5 days	31.9 days
14	Henan	Qingdao Port	7.3 days	7.4 days	14.7 days	24.5 days	31.8 days
15	Tianjin	Tianjin Port	7.5 days	7.4 days	14.9 days	24.5 days	32.0 days
16	Beijing	Tianjin Port	7.4 days	7.4 days	14.8 days	24.5 days	31.9 days
17	Hebei	Tianjin Port	7.2 days	7.4 days	14.6 days	24.5 days	31.7 days
18	Liaoning	Dalian Port	8.2 days	7.4 days	15.6 days	24.5 days	32.7 days
19	Jilin	Dalian Port	8.6 days	7.4 days	16.0 days	24.5 days	33.1 days
20	Heilongjiang	Dalian Port	8.3 days	7.4 days	15.7 days	24.5 days	32.8 days
21	Qinghai	Shanghai Port	5.9 days	7.4 days	13.3 days	24.5 days	30.4 days
22	Gansu	Shanghai Port	6.1 days	7.4 days	13.5 days	24.5 days	30.6 days
23	Ningxia	Shanghai Port	6.4 days	7.4 days	13.8 days	24.5 days	30.9 days
24	Shanxi	Shanghai Port	7.0 days	7.4 days	14.4 days	24.5 days	31.5 days
25	Shaanxi	Shanghai Port	6.8 days	7.4 days	14.2 days	24.5 days	31.3 days
26	Sichuan	Shanghai Port	7.1 days	7.4 days	14.5 days	24.5 days	31.6 days
27	Chongqing	Shanghai Port	7.3 days	7.4 days	14.7 days	24.5 days	31.8 days
28	Yunnan	Shanghai Port	8.0 days	7.4 days	15.4 days	24.5 days	32.5 days
29	Guizhou	Shanghai Port	7.7 days	7.4 days	15.1 days	24.5 days	32.2 days
30	Hunan	Shanghai Port	7.8 days	7.4 days	15.2 days	24.5 days	32.3 days
31	Hubei	Shanghai Port	7.6 days	7.4 days	15.0 days	24.5 days	32.1 days

#### 3.4.2 Distance

Distance estimations for new CPEC route are presented in Table 6. Column 4 shows the road distance in kilometres from different provinces/municipalities to Gwadar Port in Pakistan. For example, a road distance from Kashgar to Gwadar Port is 2800 kms while a distance from Zhejiang province to Gwadar port is 7870 kms. Column 5 shows the sea distance from Gwadar port in Pakistan to Port of Jebel Ali in UAE. A sea distance from Gwadar port to Port of Jebel Ali in UAE is 3365 kms. Column 6 shows the total distance including road and sea to Port of Jebel Ali in UAE from different provinces/municipalities of China. A total distance from Xinjiang to Port of Jebel Ali in UAE is 6165 kms while a total distance from Guangdong Province is 11525 kms. Column 7 shows the sea distance from Gwadar port to Hamburg port in Germany. A sea distance from Gwadar port in Pakistan to Germany is 11174 kms. Column 8 shows the total distance including road and sea to Hamburg port in Germany from different provinces/municipalities of China. A total distance from Xinjiang to Germany is 13974 kms while a total distance from Guangdong Province is 19334 kms. Details for all remaining provinces have been provided in the Table 6.

**Table 6:** New CPEC route distance.

(1) S. No	(2) Province/ Municipality	(3) Major Sea Port	(4) Road distance in Kms (From Province/ Municipality to Gwadar Port in Pakistan)	(5) Sea distance in kms (Gwadar Port to UAE)	(4+5) = (6) Total Distance (China to UAE)	(7) Sea distance kms (Gwadar Port to Germany)	(4+7) = (8) Total Distance kms (China to Germany)
1	Xinjiang	Shanghai Port	2800	3365	6165	11174	13974
2	Tibet	Shanghai Port	6122	3365	9487	11174	17296
3	Inner Mongolia	Shanghai Port	6803	3365	10168	11174	17977
4	Guangdong	Guangzhou Port	8160	3365	11525	11174	19334
5	Guangxi	Guangzhou Port	7937	3365	11302	11174	19111
6	Hainan	Guangzhou Port	8528	3365	11893	11174	19702
7	Shanghai	Shanghai Port	7914	3365	11279	11174	19088
8	Jiangsu	Shanghai Port	7624	3365	10989	11174	18798
9	Anhui	Shanghai Port	7458	3365	10823	11174	18632
10	Zhejiang	Ningbo Port	7870	3365	11235	11174	19044
11	Fujian	Ningbo Port	8180	3365	11545	11174	19354
12	Jiangxi	Ningbo Port	7640	3365	11005	11174	18814
13	Shandong	Qingdao Port	7106	3365	10471	11174	18280
14	Henan	Qingdao Port	7013	3365	10378	11174	18187
15	Tianjin	Tianjin Port	7215	3365	10580	11174	18389
16	Beijing	Tianjin Port	7143	3365	10508	11174	18317
17	Hebei	Tianjin Port	6912	3365	10277	11174	18086
18	Liaoning	Dalian Port	7844	3365	11209	11174	19018
19	Jilin	Dalian Port	8233	3365	11598	11174	19407
20	Heilongjiang	Dalian Port	7950	3365	11315	11174	19124
21	Qinghai	Shanghai Port	5673	3365	9038	11174	16847
22	Gansu	Shanghai Port	5892	3365	9257	11174	17066
23	Ningxia	Shanghai Port	6127	3365	9492	11174	17301
24	Shanxi	Shanghai Port	6693	3365	10058	11174	17867
25	Shaanxi	Shanghai Port	6545	3365	9910	11174	17719
26	Sichuan	Shanghai Port	6861	3365	10226	11174	18035
27	Chongqing	Shanghai Port	6987	3365	10352	11174	18161
28	Yunnan	Shanghai Port	7680	3365	11045	11174	18854
29	Guizhou	Shanghai Port	7364	3365	10729	11174	18538
30	Hunan	Shanghai Port	7524	3365	10889	11174	18698
31	Hubei	Shanghai Port	7281	3365	10646	11174	18455

#### 3.4.3 Cost

Table 7 presents the total cost for new CPEC route from different provinces/municipalities of China to destination ports in UAE and Germany. Column 4 shows the road cost in US dollars from different provinces/municipalities to Gwadar Port in Pakistan. For example, a road cost from Kashgar to Gwadar port is 1400 US dollars while a road cost from Zhejiang province to Gwadar port is 3935 US dollars. Column 5 shows the sea cost from Gwadar port in Pakistan to Port of Jebel Ali in UAE. A sea cost of 40 feet container from Gwadar port to Port of Jebel Ali in UAE is 1700 US dollars. Column 6 shows the total cost including road and sea to Port of Jebel Ali in UAE from different provinces/municipalities in China. A total cost of 40 feet container from Xinjiang to Port of Jebel Ali in UAE is 3100 US dollars while a total cost from Guangdong Province is 5780 US dollars. Column 8 shows the total cost including road and sea to Hamburg port in Germany from different provinces/municipalities of China. A total cost of 40 feet container from Xinjiang to Germany is 7840 US dollars while a total cost from Guangdong Province is 10520 US dollars. Details for all remaining provinces have been provided in the Table 7.

**Table 7:** New CPEC route cost.

(1) S. No	(2) Province/ Municipality	(3) Major Sea Port	(4) Road Cost in $ (From Province/ Municipality to Gwadar Port in Pakistan)	(5) Sea Cost in $ (Gwadar Port in Pakistan to UAE)	(4+5) = (6) Total cost in $ (China to UAE)	(7) Sea cost in $ (Gwadar Port in Pakistan to Germany)	(4+7) = (8) Total cost (China to Germany)
1	Xinjiang	Shanghai Port	1400	1700	3100	6440	7840
2	Tibet	Shanghai Port	3061	1700	4761	6440	9501
3	Inner Mongolia	Shanghai Port	3402	1700	5102	6440	9842
4	Guangdong	Guangzhou Port	4080	1700	5780	6440	10520
5	Guangxi	Guangzhou Port	3969	1700	5669	6440	10409
6	Hainan	Guangzhou Port	4264	1700	5964	6440	10704
7	Shanghai	Shanghai Port	3957	1700	5657	6440	10397
8	Jiangsu	Shanghai Port	3812	1700	5512	6440	10252
9	Anhui	Shanghai Port	3729	1700	5429	6440	10169
10	Zhejiang	Ningbo Port	3935	1700	5635	6440	10375
11	Fujian	Ningbo Port	4090	1700	5790	6440	10530
12	Jiangxi	Ningbo Port	3820	1700	5520	6440	10260
13	Shandong	Qingdao Port	3553	1700	5253	6440	9993
14	Henan	Qingdao Port	3507	1700	5207	6440	9947
15	Tianjin	Tianjin Port	3608	1700	5308	6440	10048
16	Beijing	Tianjin Port	3572	1700	5272	6440	10012
17	Hebei	Tianjin Port	3456	1700	5156	6440	9896
18	Liaoning	Dalian Port	3922	1700	5622	6440	10362
19	Jilin	Dalian Port	4117	1700	5817	6440	10557
20	Heilongjiang	Dalian Port	3975	1700	5675	6440	10415
21	Qinghai	Shanghai Port	2837	1700	4537	6440	9277
22	Gansu	Shanghai Port	2946	1700	4646	6440	9386
23	Ningxia	Shanghai Port	3064	1700	4764	6440	9504
24	Shanxi	Shanghai Port	3347	1700	5047	6440	9787
25	Shaanxi	Shanghai Port	3273	1700	4973	6440	9713
26	Sichuan	Shanghai Port	3431	1700	5131	6440	9871
27	Chongqing	Shanghai Port	3494	1700	5194	6440	9934
28	Yunnan	Shanghai Port	3840	1700	5540	6440	10280
29	Guizhou	Shanghai Port	3682	1700	5382	6440	10122
30	Hunan	Shanghai Port	3762	1700	5462	6440	10202
31	Hubei	Shanghai Port	3641	1700	5341	6440	10081

## 4. Results

### 4.1 Travel time

The primary goal of this research is to evaluate the differences in transportation costs, distance, and time between the traditional route and the proposed new CPEC route. The transit time for a 40-foot container on the traditional route is deducted from the transit time on the new CPEC route to get a comparative picture of the two routes. Column 4 displays the time needed to travel the conventional route, whereas column 5 displays the time needed to travel the CPEC route. Distance and time saved travelling from several Chinese provinces and municipalities to the United Arab Emirates via the new CPEC route are displayed in column 6.

**Fig 6:**
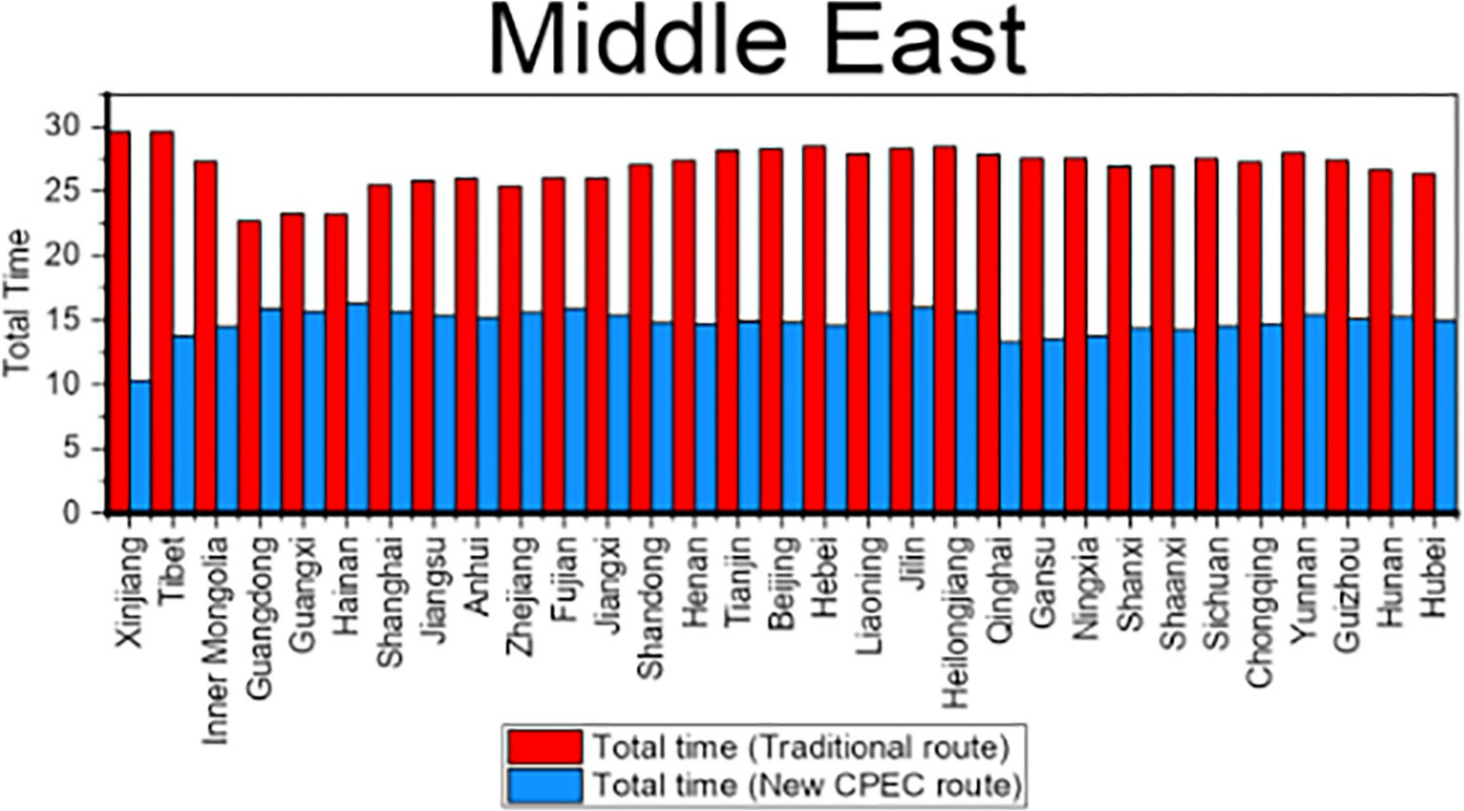
Comparison of travel time for Middle East.

**Fig 7:**
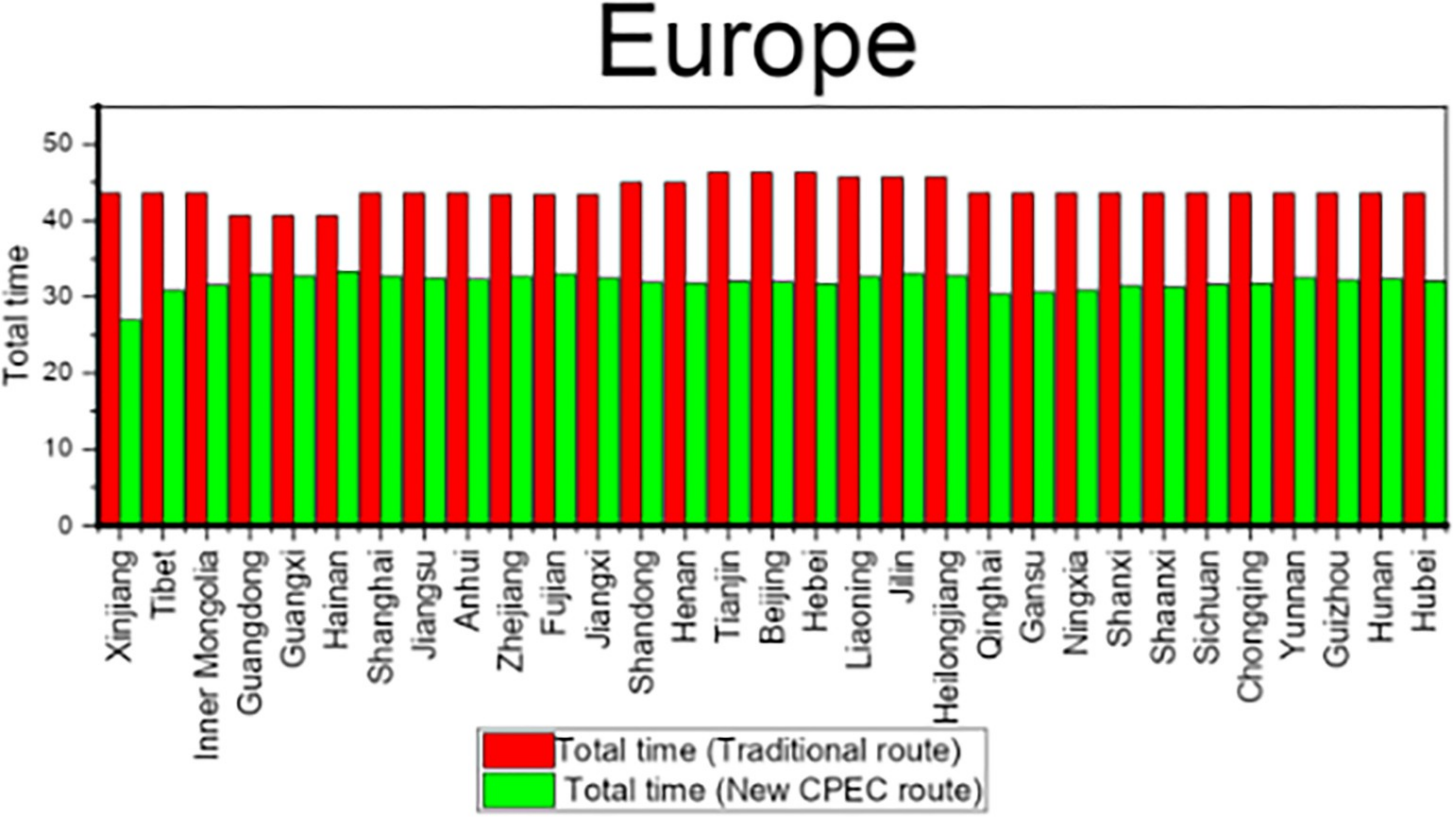
Comparison of travel time for Europe.

Results shows that the travel time from Xinjiang Province to UAE is decreased by 19 days by new CPEC route as compared to traditional route and travel time is decreased by 13 days from Tianjin municipality to UAE by new CPEC route. Likewise, column 7 shows the travel time by traditional route and column 8 shows the travel time for new CPEC route from different provinces/municipalities in China to Germany. The column 9 shows the difference and reduction in travel time by new CPEC route from different provinces/municipalities in China to Germany. The travel time from Xinjiang to Germany is decreased by 17 days by new CPEC route and travel time form Beijing to Germany is decreased by 14 days. Details for all remaining provinces have been provided in the Table 8. Fig 6 shows the comparison of travel time for Middle East while Fig 7 shows the comparison of travel time for Europe.

**Table 8:** Time difference between the traditional route and the new CPEC route.

			UAE			Germany		
(1) S. No	(2) Province/ Municipality	(3) Major Sea Port	(4) Traditional Route time	(5) New CPEC route time	(6) Difference	(7) Traditional Route time	(8) New CPEC route time	(9) Difference
1	Xinjiang	Shanghai Port	30 days	10 days	-19 days	44 days	27 days	-17 days
2	Tibet	Shanghai Port	30 days	14 days	-16 days	44 days	31 days	-13 days
3	Inner Mongolia	Shanghai Port	27 days	14 days	-13 days	44 days	32 days	-12 days
4	Guangdong	Guangzhou Port	23 days	16 days	-7 days	41 days	33 days	-8 days
5	Guangxi	Guangzhou Port	23 days	16 days	-8 days	41 days	33 days	-8 days
6	Hainan	Guangzhou Port	23 days	16 days	-7 days	41 days	33 days	-7 days
7	Shanghai	Shanghai Port	26 days	16 days	-10 days	44 days	33 days	-11 days
8	Jiangsu	Shanghai Port	26 days	15 days	-10 days	44 days	32 days	-11 days
9	Anhui	Shanghai Port	26 days	15 days	-11 days	44 days	32 days	-11 days
10	Zhejiang	Ningbo Port	25 days	16 days	-10 days	43 days	33 days	-11 days
11	Fujian	Ningbo Port	26 days	16 days	-10 days	43 days	33 days	-10 days
12	Jiangxi	Ningbo Port	26 days	-15 days	-11 days	43 days	32 days	-11 days
13	Shandong	Qingdao Port	27 days	-15 days	-12 days	45 days	32 days	-13 days
14	Henan	Qingdao Port	27 days	-15 days	-13 days	45 days	32 days	-13 days
15	Tianjin	Tianjin Port	28 days	-15 days	-13 days	46 days	32 days	-14 days
16	Beijing	Tianjin Port	28 days	-15 days	-13 days	46 days	32 days	-14 days
17	Hebei	Tianjin Port	29 days	-15 days	-14 days	46 days	32 days	-15 days
18	Liaoning	Dalian Port	28 days	-16 days	-12 days	46 days	33 days	-13 days
19	Jilin	Dalian Port	28 days	-16 days	-12 days	46 days	33 days	-13 days
20	Heilongjiang	Dalian Port	28 days	-16 days	-13 days	46 days	33 days	-13 days
21	Qinghai	Shanghai Port	28 days	-13 days	-15 days	44 days	30 days	-13 days
22	Gansu	Shanghai Port	28 days	-14 days	-14 days	44 days	31 days	-13 days
23	Ningxia	Shanghai Port	28 days	-14 days	-14 days	44 days	31 days	-13 days
24	Shanxi	Shanghai Port	27 days	-14 days	-13 days	44 days	31 days	-12 days
25	Shaanxi	Shanghai Port	27 days	-14 days	-13 days	44 days	31 days	-12 days
26	Sichuan	Shanghai Port	28 days	-15 days	-13 days	44 days	32 days	-12 days
27	Chongqing	Shanghai Port	27 days	-15 days	-13 days	44 days	32 days	-12 days
28	Yunnan	Shanghai Port	28 days	-15 days	-13 days	44 days	33 days	-11 days
29	Guizhou	Shanghai Port	27 days	-15 days	-12 days	44 days	32 days	-11 days
30	Hunan	Shanghai Port	27 days	-15 days	-11 days	44 days	32 days	-11 days
31	Hubei	Shanghai Port	26 days	-15 days	-11 days	44 days	32 days	-12days

### 4.2 Distance

When calculating the distance for a container with a length of 40 feet container along either the conventional route or the new CPEC route, the distance travelled along the traditional route is deducted from the distance along the new CPEC route.

**Fig 8:**
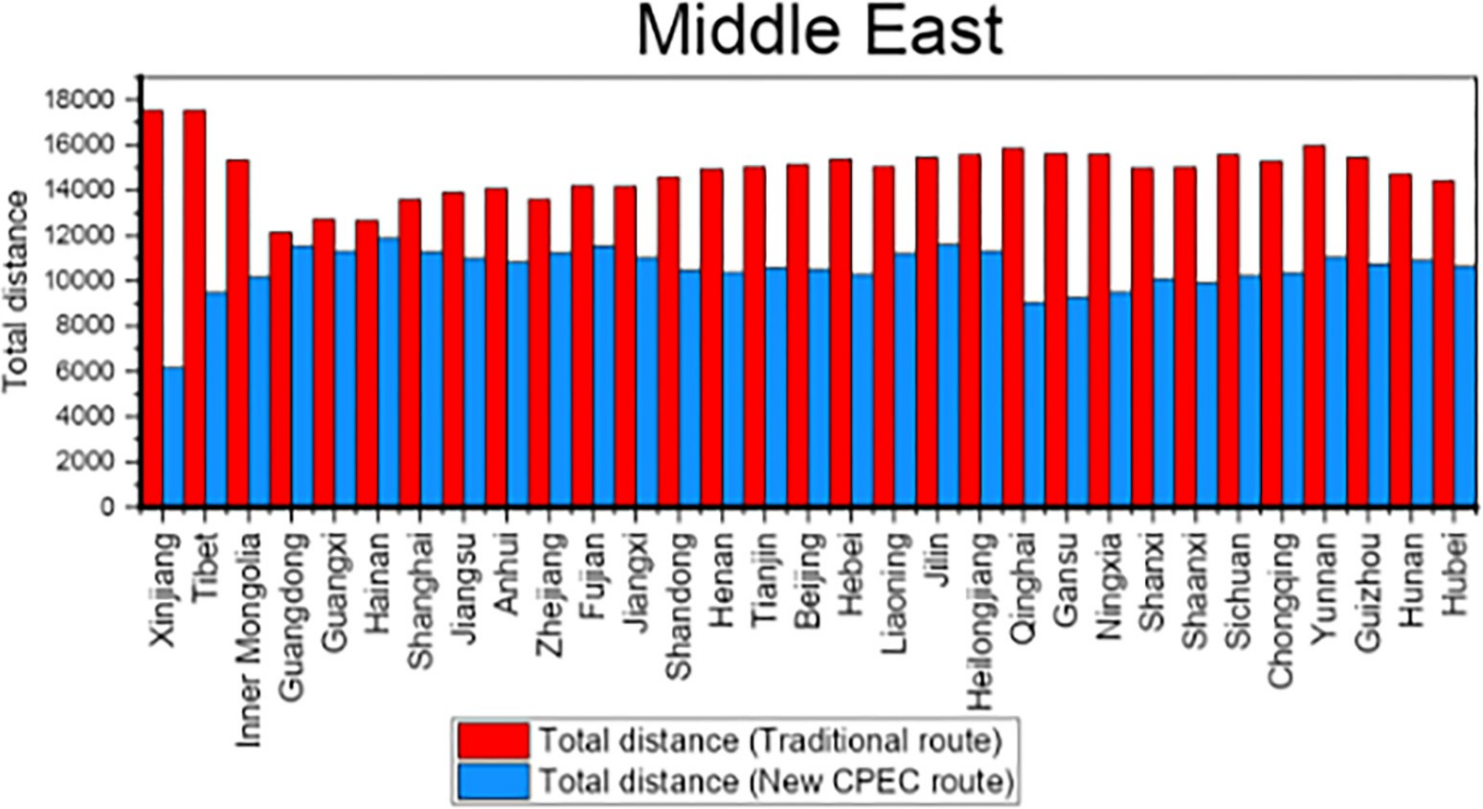
Comparison of distance for Middle East.

**Fig 9:**
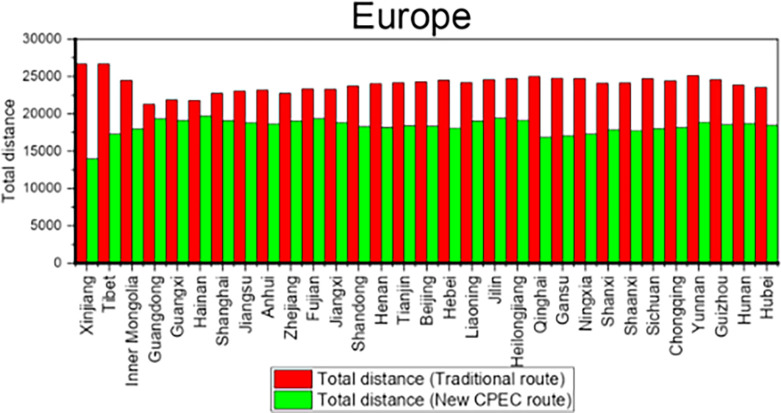
Comparison of distance for Europe.

Column 4 shows the distance for traditional route while column 5 shows the distance for new CPEC route. The column 6 shows the difference and reduction in distance by new CPEC route from different provinces/municipalities in China to UAE. Results shows that the distance from Xinjiang Province to UAE is decreased by 11381 kms by new CPEC route as compared to traditional route and distance is decreased by 4454 kms from Tianjin municipality to UAE by new CPEC route. Likewise, column 7 shows the distance by traditional route and column 8 shows the distance for new CPEC route from different provinces/municipalities in China to Germany. The column 9 shows the difference and reduction in distance by new CPEC route from different provinces/municipalities in China to Germany. The distance from Xinjiang to Germany is decreased by 12713 kms by new CPEC route and distance from Beijing to Germany is decreased by 5974 kms. Details for all remaining provinces have been provided in the Table 9. Fig 8 shows the comparison of distance for Middle East while Fig 9 shows the comparison of distance for Europe.

**Table 9:** Comparison of distance between traditional route and new CPEC route.

			UAE			Germany		
(1) S. No	(2) Province/ Municipality	(3) Major Sea Port	(4) Traditional Route distance	(5) New CPEC route distance	(6) Difference	(7) Traditional Route distance	(8) New CPEC route distance	(9) Difference
1	Xinjiang	Shanghai Port	17546 km	6165 km	-11381 km	26687 km	13974 km	-12713 km
2	Tibet	Shanghai Port	17550 km	9487 km	-8063 km	26691 km	17296 km	-9395 km
3	Inner Mongolia	Shanghai Port	15344 km	10168 km	-5176 km	24485 km	17977 km	-6508 km
4	Guangdong	Guangzhou Port	12139 km	11525 km	-614 km	21280 km	19334 km	-1947 km
5	Guangxi	Guangzhou Port	12727 km	11302 km	-1425 km	21868 km	19111 km	-2758 km
6	Hainan	Guangzhou Port	12671 km	11893 km	-778 km	21812 km	19702 km	-2111 km
7	Shanghai	Shanghai Port	13601 km	11279 km	-2322 km	22742 km	19088 km	-3654 km
8	Jiangsu	Shanghai Port	13908 km	10989 km	-2919 km	23049 km	18798 km	-4251 km
9	Anhui	Shanghai Port	14063 km	10823 km	-3240 km	23204 km	18632 km	-4572 km
10	Zhejiang	Ningbo Port	13609 km	11235 km	-2374 km	22751 km	19044 km	-3707 km
11	Fujian	Ningbo Port	14201 km	11545 km	-2656 km	23343 km	19354 km	-3989 km
12	Jiangxi	Ningbo Port	14171 km	11005 km	-3166 km	23313 km	18814 km	-4499 km
13	Shandong	Qingdao Port	14588 km	10471 km	-4118 km	23730 km	18280 km	-5450 km
14	Henan	Qingdao Port	14924 km	10378 km	-4547 km	24066 km	18187 km	-5879 km
15	Tianjin	Tianjin Port	15034 km	10580 km	-4454 km	24176 km	18389 km	-5787 km
16	Beijing	Tianjin Port	15149 km	10508 km	-4641 km	24291 km	18317 km	-5974 km
17	Hebei	Tianjin Port	15372 km	10277 km	-5095 km	24514 km	18086 km	-6428 km
18	Liaoning	Dalian Port	15053 km	11209 km	-3844 km	24193 km	19018 km	-5175 km
19	Jilin	Dalian Port	15438 km	11598 km	-3840 km	24578 km	19407 km	-5171 km
20	Heilongjiang	Dalian Port	15582 km	11315 km	-4267 km	24722 km	19124 km	-5598 km
21	Qinghai	Shanghai Port	15855 km	9038 km	-6817 km	24996 km	16847 km	-8149 km
22	Gansu	Shanghai Port	15611 km	9257 km	-6354 km	24752 km	17066 km	-7686 km
23	Ningxia	Shanghai Port	15585 km	9492 km	-6093 km	24726 km	17301 km	-7425 km
24	Shanxi	Shanghai Port	14973 km	10058 km	-4915 km	24114 km	17867 km	-6247 km
25	Shaanxi	Shanghai Port	15022 km	9910 km	-5112 km	24163 km	17719 km	-6444 km
26	Sichuan	Shanghai Port	15565 km	10226 km	-5339 km	24706 km	18035 km	-6671 km
27	Chongqing	Shanghai Port	15297 km	10352 km	-4945 km	24438 km	18161 km	-6277 km
28	Yunnan	Shanghai Port	15985 km	11045 km	-4940 km	25126 km	18854 km	-6272 km
29	Guizhou	Shanghai Port	15436 km	10729 km	-4707 km	24577 km	18538 km	-6039 km
30	Hunan	Shanghai Port	14715 km	10889 km	-3826 km	23856 km	18698 km	-5158 km
31	Hubei	Shanghai Port	14428 km	10646 km	-3782 km	23569 km	18455 km	-5114 km

### 4.3 Cost

**Fig 10:**
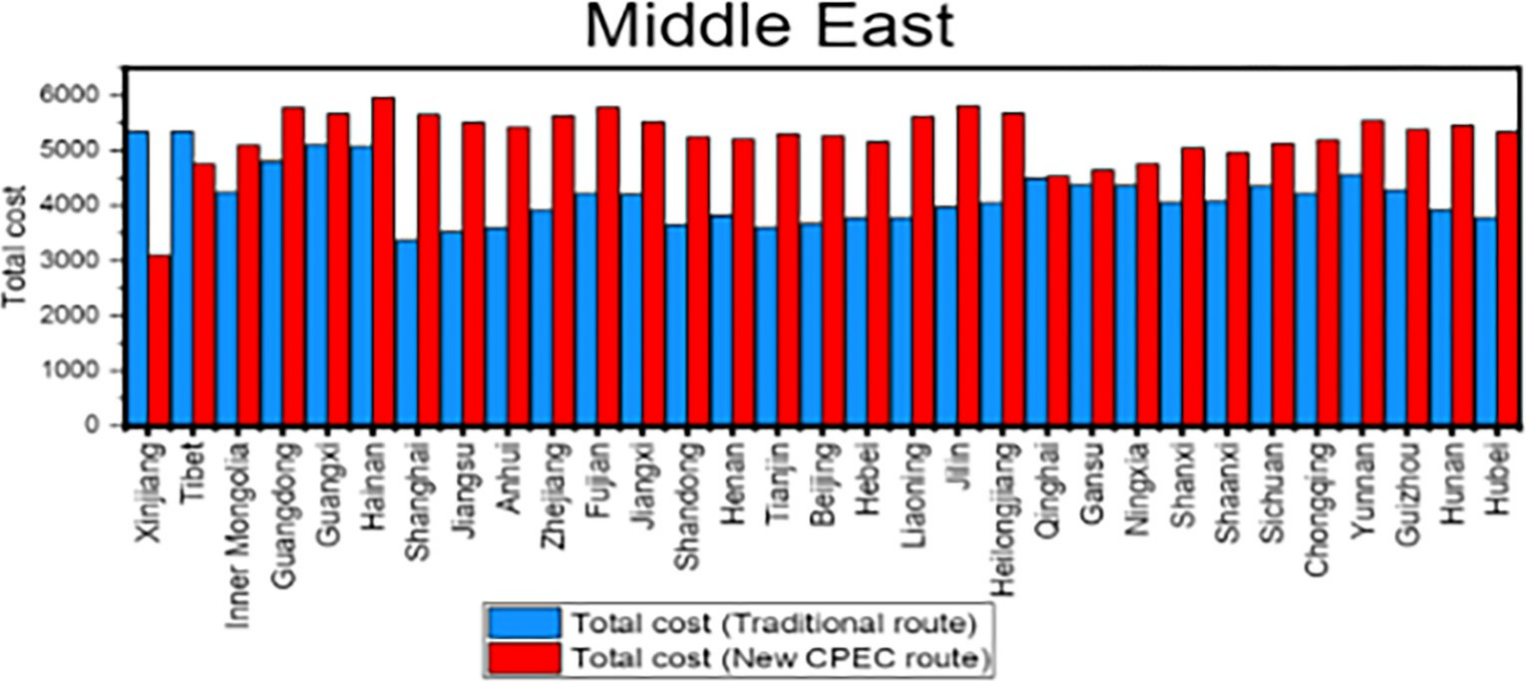
Comparison of cost for Middle East.

**Fig 11:**
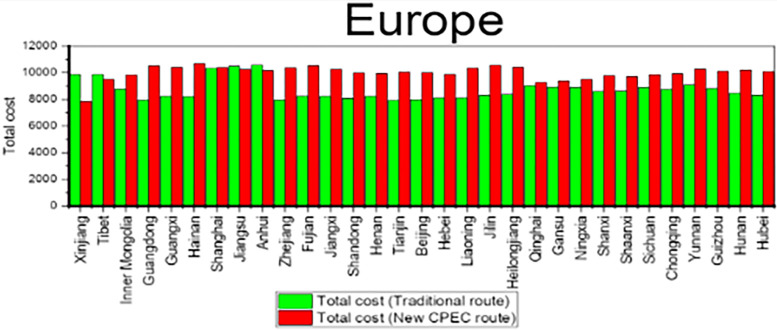
Comparison of cost for Europe.

To compare the cost for a 40-foot container between traditional route and new CPEC route, cost for the traditional route is subtracted from cost for new CPEC route. Column 4 shows the cost for traditional route while column 5 shows the cost for new CPEC route. The column 6 in Table 9 shows the difference in cost by new CPEC route from different provinces/municipalities in China to UAE. Results shows that the cost from Xinjiang Province to UAE is decreased by 2243 US dollars by new CPEC route as compared to traditional route and cost is increased by 1695 US dollars from Tianjin municipality to UAE by new CPEC route. Likewise, column 7 shows the cost by traditional route and column 8 shows the cost for new CPEC route from different provinces/municipalities in China to Germany. The column 9 shows the difference in cost by new CPEC route from different provinces/municipalities in China to Germany. The cost from Xinjiang to Germany is decreased by 2041 US dollars by new CPEC route and distance from Beijing to Germany is increased by 2020 US dollars. Details for all remaining provinces have been provided in the Table 10. Fig 10 shows the comparison of cost for Middle East while Fig 11 shows the comparison of cost for Europe.

**Table 10:** Comparison of cost between traditional route and new CPEC route.

			UAE			Germany		
(1) S. No	(2) Province/ Municipality	(3) Major Sea Port	(4) Traditional Route cost	(5) New CPEC route cost	(6) Difference	(7) Traditional Route cost	(8) New CPEC route cost	(9) Difference
1	Xinjiang	Shanghai Port	5343 $	3100 $	-2243 $	9881 $	7840 $	-2041 $
2	Tibet	Shanghai Port	5345 $	4761 $	-584 $	9883 $	9501 $	-382 $
3	Inner Mongolia	Shanghai Port	4242 $	5102 $	860 $	8780 $	9842 $	1062 $
4	Guangdong	Guangzhou Port	4811 $	5780 $	969 $	7949 $	10520 $	2571 $
5	Guangxi	Guangzhou Port	5105 $	5669 $	564 $	8243 $	10409 $	2166 $
6	Hainan	Guangzhou Port	5077 $	5964 $	887 $	8215 $	10704 $	2489 $
7	Shanghai	Shanghai Port	3371 $	5657 $	2287 $	10344 $	10397 $	54 $
8	Jiangsu	Shanghai Port	3524 $	5512 $	1988 $	10497 $	10252 $	-245 $
9	Anhui	Shanghai Port	3602 $	5429 $	1828 $	10575 $	10169 $	-406 $
10	Zhejiang	Ningbo Port	3923 $	5635 $	1712 $	7961 $	10375 $	2414 $
11	Fujian	Ningbo Port	4219 $	5790 $	1571 $	8257 $	10530 $	2273 $
12	Jiangxi	Ningbo Port	4204 $	5520 $	1316 $	8242 $	10260 $	2018 $
13	Shandong	Qingdao Port	3655 $	5253 $	1599 $	8077 $	9993 $	1917 $
14	Henan	Qingdao Port	3823 $	5207 $	1384 $	8245 $	9947 $	1702 $
15	Tianjin	Tianjin Port	3613 $	5308 $	1695 $	7935 $	10048 $	2113 $
16	Beijing	Tianjin Port	3670 $	5272 $	1602 $	7992 $	10012 $	2020 $
17	Hebei	Tianjin Port	3782 $	5156 $	1375 $	8104 $	9896 $	1793 $
18	Liaoning	Dalian Port	3785 $	5622 $	1837 $	8107 $	10362 $	2255 $
19	Jilin	Dalian Port	3978 $	5817 $	1839 $	8300 $	10557 $	2257 $
20	Heilongjiang	Dalian Port	4050 $	5675 $	1626 $	8372 $	10415 $	2044 $
21	Qinghai	Shanghai Port	4498 $	4537 $	39 $	9036 $	9277 $	241 $
22	Gansu	Shanghai Port	4376 $	4646 $	271 $	8914 $	9386 $	473 $
23	Ningxia	Shanghai Port	4363 $	4764 $	401 $	8901 $	9504 $	603 $
24	Shanxi	Shanghai Port	4057 $	5047 $	990 $	8595 $	9787 $	1192 $
25	Shaanxi	Shanghai Port	4081 $	4973 $	892 $	8619 $	9713 $	1094 $
26	Sichuan	Shanghai Port	4353 $	5131 $	778 $	8891 $	9871 $	980 $
27	Chongqing	Shanghai Port	4219 $	5194 $	975 $	8757 $	9934 $	1177 $
28	Yunnan	Shanghai Port	4563 $	5540 $	978 $	9101 $	10280 $	1180 $
29	Guizhou	Shanghai Port	4288 $	5382 $	1094 $	8826 $	10122 $	1296 $
30	Hunan	Shanghai Port	3928 $	5462 $	1535 $	8466 $	10202 $	1737 $
31	Hubei	Shanghai Port	3784 $	5341 $	1557 $	8322 $	10081 $	1759 $

## 5. Conclusion

The main aim of this study is to examine and compare the travel time, distance, and cost of 40 feet container between traditional route and new CPEC route. Transportation plays significant role in the transfer of raw material and finish goods. A good transportation infrastructure enables safe, faster, and low-cost movement of goods. The launch of the China-Pakistan Economic Corridor could improve the international transport network between China and Pakistan, thus plummeting the dependence of the China on the traditional route through the Strait of Malacca. The CPEC will improve the connectivity between Pakistan and China through construction of various transportation infrastructure projects and upgradation projects. The CPEC has the potential to serve as the China-Middle East and China-Europe alternative trading route.

China requires an another, safe and short trading route with Middle East and Europe. In this connection, China and Pakistan will not only save millions of dollars in transportation cost but can also save travel time and shorter distance. The China-Pakistan Economic Corridor is short and a substitute route that connects Xinjiang Province of China to the Gwadar seaport in Pakistan by developing a better road transportation infrastructure network and upgradation of railway network. Gwadar is a deep-water seaport located at the mouth of the Persian Gulf, near the Strait of Hormuz. Gwadar seaport is highly attractive for China to handle sea transport challenges and link Western China to the world through regional and economic connectivity.

In this study, the transportation costs, travel time, and distance were estimated and compared for both the new CPEC route and the traditional route. These two routes encompass both land and sea transportation. A detailed description of the variables related to travel time, cost, and distance was discussed in the paper. Due to the nature of the available data, it was not feasible to employ advanced econometric estimation techniques. The collected data was scattered and discrete. Given these limitations, this research employed a simple statistical analysis using MS Excel. Secondary data on travel time, cost, and distance was collected from various online sources, including shipping corporations, Google Maps, and transportation companies. The gathered data was then cleaned and systematically arranged for analysis in MS Excel.

Through widespread examination based on the travel time, distance, and cost of a general product from China to Middle East and Europe, the substantial benefits of the China-Pakistan Economic Corridor over the traditional route are proved. Unambiguously, we find that the new CPEC route is advantageous when the travel time requirement is inflexible, while the traditional route could still be beneficial to some coastline provinces in terms of cost. In short, the new CPEC route is found to be the most necessary trading route alternative in terms of travel time and distance for all the provinces/municipalities of China, while the new CPEC route could be preferable in terms of cost for the provinces located in Western China. This paper analysed three indicators namely time, distance, and cost to compare the pros and cons of traditional and new CPEC route. Out of the three indicators, in case of the new CPEC route, time and distance are substantially lower for all the provinces as compared to the traditional route. However, the evidence presented by this research suggests that although the cost of road transportation via the new CPEC route is lower for Xinjiang province, the cost of road transportation for rest of provinces has increased. Therefore, the new CPEC route is unfavourable in terms of transportation cost for the provinces/municipalities in Eastern China.

The findings of this study shows that China will get a short and safe route for all provinces/municipalities to trade with Middle East and Europe. The travel time will reduce by 10 to 20 days approximately from provinces/municipalities in China to Middle East and Europe. The distance will reduce by 3000 to 10000 kms approximately from provinces/municipalities in China to Middle East and Europe. The transport cost will reduce by about 2000 dollars from Xinjiang (Western China) to Middle East and Europe.

The existing literature does not sufficiently address the costs and benefits of the traditional and new CPEC routes between China, Middle East, and Europe. To our knowledge, very few studies have statistically analysed the various effects of the CPEC on the trade route choices in various Chinese provinces and municipalities. In this study, we statistically analysed three variables—time, distance, and cost—to evaluate the advantages of the old and new CPEC routes. These comparisons have significant policy implications for both government decision-makers and business stakeholders.

## 6. Limitations

The traditional route and new CPEC route consist of road and sea. It is quite easy to calculate the sea transportation cost and travel time, however it is difficult for road transportation because local transporters charge differently in Pakistan and China. Therefore, average per kilometre cost of 0.5 US dollars is used to get the reliable results for both traditional and new CPEC route. The transportation cost of general product of 40-foot container provided by shipping companies (Hapag-Llyod) is normally effective for one month. The cost may change subject to different factors like oil prices, supply, and demand. CPEC project is not fully functional, so it is difficult to get the exact data of travel time and cost related to road transport.

## Supporting information

S1 Data set (XLSX)
